# Bovine Serum Albumin Effect on Collapsing PNIPAM Chains in Aqueous Solutions: Spin Label and Spin Probe Study

**DOI:** 10.3390/polym16101335

**Published:** 2024-05-09

**Authors:** Georgii A. Simenido, Ekaterina M. Zubanova, Evgenii A. Ksendzov, Sergei V. Kostjuk, Peter S. Timashev, Elena N. Golubeva

**Affiliations:** 1 Faculty of Chemistry, Lomonosov Moscow State University, 119991 Moscow, Russia; kate_zub@mail.ru (E.M.Z.); timashev_p_s@staff.sechenov.ru (P.S.T.); legol@mail.ru (E.N.G.); 2 Research Institute for Physical Chemical Problems of the Belarusian State University, 220006 Minsk, Belarus; zheniy-98@tut.by (E.A.K.); kostjuks@bsu.by (S.V.K.); 3 Faculty of Chemistry, Belarusian State University, 220006 Minsk, Belarus; 4 Institute for Regenerative Medicine, Sechenov First Moscow State Medical University, 119991 Moscow, Russia

**Keywords:** thermoresponsive polymers, electron paramagnetic resonance, spin probe, spin label, nitroxides, coil to globule, inhomogeneities, polymer–protein interaction, bovine serum albumin

## Abstract

The influence of bovine serum albumin (BSA) on collapsing poly(*N*-isopropylacrylamide) (PNIPAM) chains was studied with turbidimetry and spin probe and spin label electron paramagnetic resonance spectroscopy. An increased ratio of collapsed chains in aqueous solutions in the narrow temperature region near the LCST appeared in the presence of 2.5–10 wt% BSA. The spin probe EPR data indicate that the inner cavities of the BSA dimers are probably responsive to the capture of small hydrophobic or amphiphilic molecules, such as TEMPO nitroxyl radical. The observed features of the structure and dynamics of inhomogeneities of aqueous PNIPAM-BSA solutions, including their mutual influence on the behavior of the polymer and protein below the LCST, should be considered when developing and investigating PNIPAM-based drug delivery systems.

## 1. Introduction

Smart or stimuli-responsive polymers remain attractive to researchers due to the ability to control their physicochemical properties in solutions by different stimuli, such as temperature [1], ionic strength [2], pH [3], light irradiation [4], and biological molecules [5], etc. According to the architecture of polymeric systems (e.g., solutions, microgels, or micelles), a response to an external stimulus may lead to changes in the swelling degree or solubility up to macroscopic phase separation [6]. This non-linear response usually comes from the collapse of polymer chains due to the coil-to-globule phase transition. The temperature of the coil-to-globule transition is one of the main characteristics of stimuli-responsive polymers. Depending on the stimuli-responsive polymer’s structure and solvent nature, polymer–solvent systems may be characterized by an upper critical solution temperature (UCST) and/or lower critical solution temperature (LCST). Polymers with an LCST are usually soluble in polar solvents below the critical temperature and undergo polymer chain collapse when heated to above the LCST. However, for multi-responsive polymers, the addition of other stimuli may lead to changes in the LCST, allowing control over the polymer system through irradiation or changes in the chemical composition of the solution, including its pH, ionic strength, etc. [7,8,9,10].

A controllable response allows for the use of smart polymeric materials in biomedicine and tissue engineering as drug and biomolecules delivery systems, coatings, and hydrogels for 2D and 3D cell structures [11,12,13,14]. In the first case, there are two mechanisms of drug release depending on the architecture of the polymer matrix. The release of a loaded drug may occur through the hydration of a polymer chain below the LCST, followed by swelling and dissolution [15], or by the collapse and shrinkage of the polymer hydrogel, leading to a rapid drug release [16]. Applications of smart polymers as coatings for cell structures are based on the different adhesion of cells to the polymer surfaces at different temperatures. The surfaces of stimuli-responsive polymers are adhesive to cells above the LCST, leading to cellular reproduction and growth in the cultural environment [17]. Below the LCST, the polymer material swells or dissolves due to the phase transition, which causes cell sheet detachment and preserves the extracellular matrix (ECM) structure. These approaches allow for the production of cell structures with ECMs close to those found in native tissues, which can be applied in the construction of artificial organs.

The most popular smart polymer is thermoresponsive poly-*N*-isopropylacrylamide (PNIPAM), which was first synthesized in 1968 and has an LCST of 32 °C in aqueous solutions [18]. It contains hydrophobic alkyl fragments and hydrophilic amide groups in the polymer chain links. In aqueous solutions below the LCST, the chains are hydrated due to intermolecular hydrogen bonds between the water molecules and the donor atoms of the amide group. Breaking these H-bonds upon heating results in the prevalence of hydrophobic interactions, the formation of intramolecular H-bonds, and the collapse of polymer chains. The conditions and the rate of the collapse are determined by hydrophobic–hydrophilic balance. This balance may be controlled by the polymer and solution composition, e.g., the use of copolymers with hydrophobic or hydrophilic monomers, adding components capable of hydration, etc., to the solutions.

PNIPAM and its copolymers may be used in complex drug delivery systems or as coatings for cell sheets. In all described biomedical applications, the polymeric matrix is located in multicomponent aqueous solutions that contain different ions, amino acids, and carbohydrates in the cell culture or proteins in the blood and ECM. These components may act as chemical stimuli on the LCST and can influence the rate and equilibrium of the polymer collapse by changing the hydrophobic–hydrophilic balance. For example, ions may decrease the LCST [19,20] due to the salting out of the polymer by solvation with adjacent water molecules. Some proteins, e.g., myoglobin, may compete with PNIPAM for water molecules, leading to faster dehydration of the polymer chains during polymer collapse and resulting in the LCST decreasing [21].

No systematic study of the interactions between model proteins and PNIPAM has been performed, so, in present study, the effect of bovine serum albumin (BSA), which is a model for blood serum components, on the collapse of PNIPAM polymer chains was studied by turbidimetry and spin label and spin probe EPR spectroscopy. EPR spectroscopy is a well-proven approach to investigate the dynamics of polymer chains and the micropolarity and microviscosity of inhomogeneities in polymer solutions. In the case of the spin label technique, a stable paramagnetic radical—usually a nitroxide radical—is bound to the polymer chain via a chemical covalent bond, e.g., an amide bond. The EPR spectra of spi*N*-labeled polymers allow for overseeing the dynamics of the polymer chains by determination of the rotational correlation time (*t*_corr_), denoted as the time of rotation by one radian. In the spin probe method, a stable paramagnetic radical is freely distributed in a polymer solution and may be captured by the collapsing polymer chains when the phase transition occurs. Previously, the spin probe technique has been implemented to study the structure and dynamics of inhomogeneities in a number of polymers [22,23,24], including PNIPAM and its copolymers [25,26], using nanoscopic amphiphilic nitroxide probe (2,2,6,6-tetramethylpiperidi*N*-1-yl)oxyl (TEMPO). This technique affords the observation of the formation of nanoscale inhomogeneities in polymer aqueous solutions below the LCST, estimated by differential scanning calorimetry (DSC), and to study the dynamics of these inhomogeneities and their micropolarity. In the present study, we used the same spin probe technique to investigate PNIPAM-BSA solutions with a combination of the spin label approach and turbidimetry to determine how and under what conditions BSA may influence the collapse of PNIPAM polymer chains. Promoting PNIPAM chain collapse by BSA near the LCST was revealed through turbidimetry and EPR spectroscopy. The obtained peculiarities in the structure and dynamics of the inhomogeneities in aqueous PNIPAM-BSA solutions should be considered when developing and studying PNIPAM-based drug delivery systems.

## 2. Materials and Methods

### 2.1. Substances

Stable radical (2,2,6,6-tetramethylpiperidi*N*-1-yl)oxyl (TEMPO), copper(II) chloride dihydrate CuCl_2_·2H_2_O, succinic anhydride (≥99%), hydroxylamine hydrochloride (98.0%), isopropylamine (≥97%), benzene (anhydrous, 99.8%), and 4-amino-(2,2,6,6-tetramethylpiperidi*N*-1-yl)oxyl (4-amino-TEMPO) (97%), purchased from Sigma-Aldrich (Burlington, MA, USA), were used without further purification. *N*-isopropylacrylamide (NIPAM) (97%, Sigma Aldrich, Burlington, MA, USA) was recrystallized using a 30% solution in hexane and dried in vacuum at r.t. and 1 mmHg. 1-Butanol (≥99.5%, Roth, Karlsruhe, Germany) was used as received. Azobisisobutyronitrile (AIBN) (98%, Biolar, Olaine, Latvia) was recrystallized using a solution in methanol and dried in vacuum at r.t. and 1 mmHg. Acryloyl chloride (98%, Acros Organics, Geel, Belgium) was distilled under argon, stabilized with 0.1% BHT, and stored at −30 °C. Diethyl ether (for analysis), acetone (for analysis), ethyl acetate (pure), and hexane (pure) were purchased from Ekos-1 (Moscow, Russia) and used without purification. Tetrahydrofuran (THF) (LiChrosolv^®^, >99.9%), purchased from Merck (Darmstadt, Germany), was used as received. Dichloromethane (for synthesis, Ekos-1, Moscow, Russia) was treated with 98% sulfuric acid, then washed with water and dried with anhydrous CaCl_2_. It was refluxed and distilled twice using CaH_2_. Triethylamine (for synthesis, Sigma Aldrich, Burlington, MA, USA) was refluxed and distilled twice using CaH_2_. 1,4-Dioxane was distilled with NaOH, and then refluxed and distilled using sodium metal under argon. Dimethylformamide (DMF) (for HPLC), purchased from Carlo Erba (Emmendingen, Germany), was distilled under vacuum. LiBr (99%, Acros Organics, Geel, Belgium) was used as received. Bovine serum albumin (BSA), purchased from PanEco Ltd. (Moscow, Russia), was used without further purification.

### 2.2. PNIPAM Synthesis

Poly(*N*-isopropylacrylamide) (PNIPAM) was prepared by the free-radical polymerization of NIPAM in benzene using AIBN as an initiator, as described in [25]. The product had M_n_ = 175.5 kDa and Ð = 4.3, which were determined by SEC.

### 2.3. SL-PNIPAM Synthesis

**Scheme 1:**
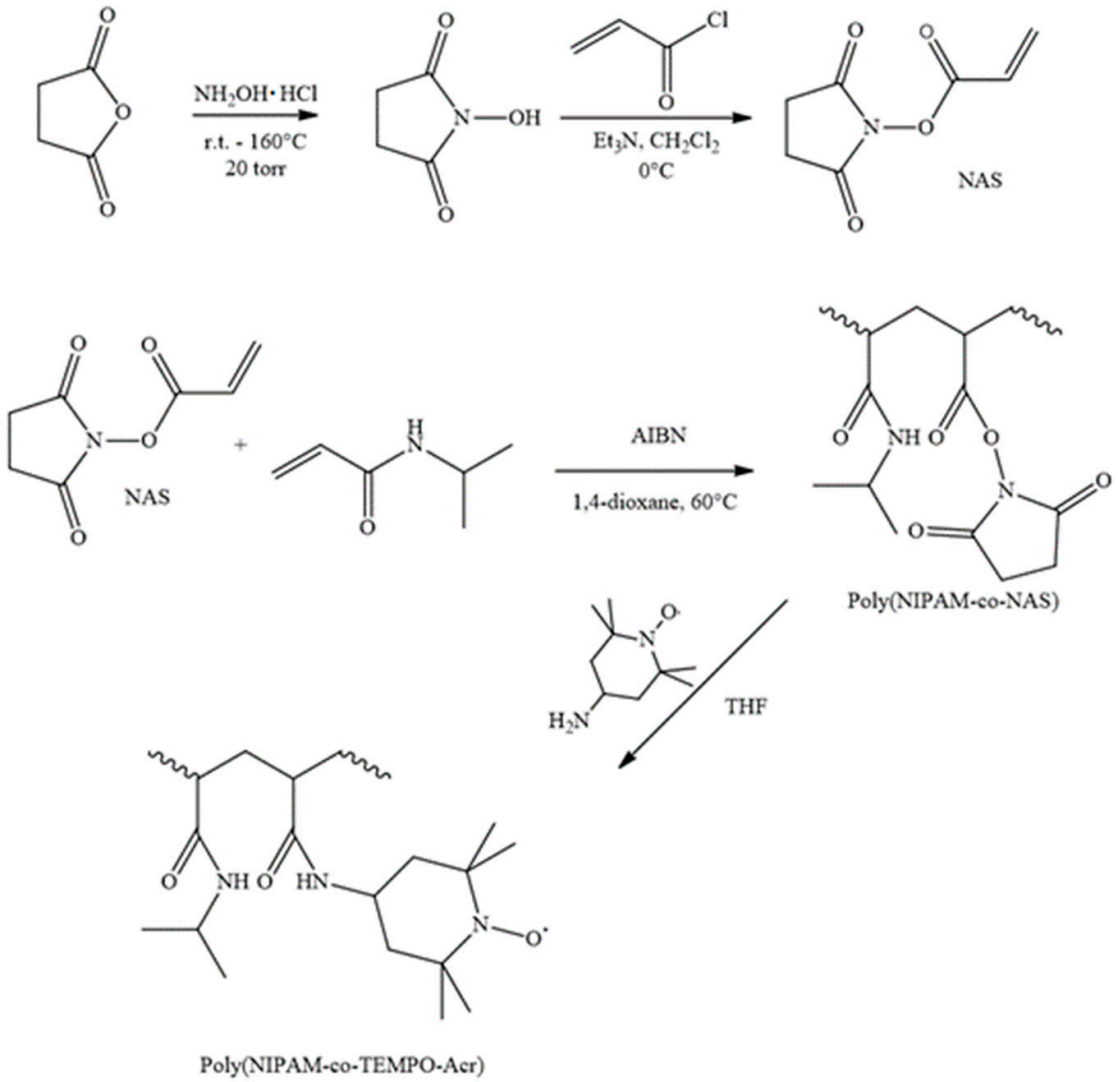
Synthesis of SL-PNIPAM.

Spi*N*-labeled PNIPAM (SL-PNIPAM) was synthesized by postpolymerization modification of the NIPAM/*N*-acryloxysuccinimide (NAS) copolymer using 4-amino-TEMPO, as proposed in [27]. Details of the synthesis steps (see Scheme 1) are given below.

#### 2.3.1. Synthesis of *N*-Hydroxysuccinimide [28]

Succinic anhydride (16.2 g, 0.162 mol) was mixed with hydroxylamine hydrochloride (11.3 g, 0.163 mol), and the mixture was heated to 125 °C at 20 mmHg under stirring. The temperature was raised to 160 °C over 2 h. After cooling to r.t., 65 mL of ether was added to amber oil residue, and the mixture was sonicated. The filtered solid substance was recrystallized using 65 mL of 1-butanol. Crystals of *N*-hydroxysuccinimide were washed with cold 1-butanol (10 mL) and ether (150 mL) and dried in vacuum at 1 mmHg and r.t. Another crystallization was performed using 65 mL of ethyl acetate, and the crystals were dried in a similar manner. The overall yield was 6.63 g (36%).

#### 2.3.2. Synthesis of *N*-Acryloxysuccinimide (NAS)

*N*-Hydroxysuccinimide (0.821 g, 7.13 mmol) was dissolved in a mixture of CH_2_Cl_2_ (11.4 mL) and triethylamine (1.26 mL, 9.04 mmol). The solution was cooled to 0 °C, and acryloyl chloride (0.57 mL, 6.6 mmol) was added. After 40 min, the reaction mixture was diluted with CH_2_Cl_2_ and thrice washed with 5% NaHCO_3_. Then, the solution was dried using water with anhydrous Na_2_CO_3_ and using solvent in vacuum at 1 mmHg and r.t. The overall yield was 1.06 g (95%).

#### 2.3.3. Copolymerization of NIPAM and NAS

NIPAM (5.00 g, 44.2 mmol), NAS (0.149 g, 0.882 mmol), and AIBN (36.5 mg, 0.222 mmol) were placed in the Schlenk reactor, thrice vacuumed, and filled with argon. Then, 22 mL of 1,4-dioxane as a solvent was added, and the solution was bubbled with argon for 30 min. The closed reactor was then put into a preheated oil bath (60 °C), and the solution was stirred at this temperature for 24 h. Then, the reaction mixture was cooled with liquid nitrogen, heated to r.t., and the solvent was rotary-evaporated. The residue was thrice precipitated using an acetone solution (50 mL of acetone) into 500 mL of hexane. The product P(NIPAM-co-NAS) had M_n_ = 101 kDa and Ð = 3.3, which were determined by SEC.

#### 2.3.4. TEMPO-Modification of P(NIPAM-co-NAS)

P(NIPAM-co-NAS) (1 g) was dissolved in 10 mL of anhydrous THF, and 6 mg of 4-amino-TEMPO was added. The solution was stirred for 21 h at r.t. until isopropylamine (0.15 mL) was added. After 4 h, the mixture was poured into hexane. The product was precipitated two more times from acetone to hexane and dried in vacuum at 40 °C and 1 mmHg. The product had M_n_ = 101 kDa and Ð = 3.3, which were determined by SEC. The SEC curves for P(NIPAM-co-NAS) and SL-PNIPAM are listed in the Supplementary Materials (Figure S1). The prepared spi*N*-labeled polymer was purified with the impurity of unbound 4-amino-TEMPO using dialysis tubing with a molecular weight cut-off of 500 Da until the change in the number of paramagnetic particles ceased. The content of the label, determined by EPR spectroscopy, was one 4-amino-TEMPO unit per 4500 NIPAM units.

### 2.4. Size Exclusion Chromatography (SEC)

SEC was carried out using the Ultimate 3000 Thermo Scientific chromatographic system (Thermo Scientific Dionex, Regensburg, Germany), equipped with a PLgel pre-column guard (size of 7.5 × 50 mm^2^, particle size of 5 μm, Agilent Technologies, Santa Clara, CA, USA) and a PLgel MIXED-C column (size of 7.5 × 300 mm^2^, particle size of 5 μm, Agilent Technologies, Santa Clara, CA, USA) thermostated at 50 °C. The refractive index detector was thermostated at 40 °C. Elution was performed in the isocratic mode with DMF containing 0.1 M LiBr at a flow rate of 1 mL/min. The molar masses and polydispersity indexes of the polymers were calculated using the Chromeleon 7 program (Thermo Scientific Dionex, Regensburg, Germany) based on polymethylmethacrylate standards (ReadyCal Kit, PSS GmbH, Mainz, Germany), with M_w_/M_n_ ≤ 1.05.

### 2.5. Solution Preparation

#### 2.5.1. PNIPAM/BSA Solutions

Aqueous solutions containing 1, 5, and 10 wt% PNIPAM and 0, 2.5, 5, and 10 wt% BSA were prepared by dissolving predetermined amounts of PNIPAM and albumin in distilled water and equilibrating them for 24 h at 4 °C until complete dissolution of the polymer occurred. A predetermined amount of TEMPO aqueous solution was added to obtain 0.5 mM of TEMPO solution for EPR spin probe experiments.

#### 2.5.2. SL-PNIPAM/BSA Solutions

SL-PNIPAM/BSA solutions were prepared by adding predetermined amounts of BSA and distilled water to the 10 wt% SL-PNIPAM solution after dialysis to achieve solutions with w(SL-PNIPAM)/w(BSA) ratios equal to 4:1, 2:1, and 1:2, as shown in Table A1.

### 2.6. Turbidimetry (Step-Wise Regime)

The polymer solutions were placed in a homemade 1 mm thermostated cuvette. A nichrome wire was wrapped around the cuvette and used as a heater. The temperature was monitored using a DS18B20 (Analog Devices, Wilmington, MA, USA) temperature sensor attached to the back wall of the cuvette. The heating was controlled by an Arduino Uno (Arduino, Monza, Italy) microcontroller using a PID algorithm. The temperature control accuracy was about 0.3 °C. Transmittance measurements were obtained using a UV-Vis spectrophotometer, the UV-2401PC (Shimadzu, Tokyo, Japan), at 405 nm. The measurements were conducted at 25–35 °C in 1 °C intervals. At each step, the system was equilibrated until the transmittance fluctuations became less than 0.5%. The cloud point temperature (*T*_cp_) was determined as the temperature corresponding to 90% transmittance. After 35 °C, when the LCST was clearly passed and the transmittance was low, an additional measurement was taken at 40 °C to ensure that the transmittance was about zero.

### 2.7. EPR Spectroscopy

The solutions were placed in 2 mm glass tubes, which were then sealed to prevent the evaporation of water. The EPR spectra were recorded using the X-band spectrometer Bruker EMX-500 (Bruker, Karlsruhe, Germany). The temperature dependence of the spectra was recorded using a Bruker temperature control unit (accuracy 0.5 °C). Each sample was held before recording at a certain temperature for 5 min for equilibration. Typical parameters of the spectra recording were a microwave power of 0.8 mW, a modulation amplitude of 0.04 mT, and a sweep width of 8 mT. Suppression of the fast spin probe EPR signal in the solution was achieved by adding 10 mg of CuCl_2_·2H_2_O to 0.5 mL of the polymer solution, as recommended in [25].

### 2.8. EPR Spectra Treatment and Simulation

The amplitudes and linewidths of the spectral lines were measured using the EsrD program (Chemistry Faculty of Lomonosov Moscow State University) [29]. Spectra with a poor signal-to-noise ratio were smoothed using the 5 pts FFT smoothing method for better visual appearance. All spectra simulations were performed using homemade scripts for the EasySpin (v. 5.2.35) ope*N*-source MATLAB toolkit [30]. The ‘chili’ function in EasySpin was used to simulate the TEMPO and SL-PNIPAM spectra. The slow-motion ‘chili’ model is based on the Schneider–Freed theory [31,32], solving equations for slowly tumbling nitroxides. The anisotropic values of the spin Hamiltonian parameters (the **g**-tensor and the hyperfine coupling tensor, usually denoted as the **A**-tensor) were averaged to obtain the *g*_iso_ and *a*_iso_ values. The rotational correlation time tensor (*t*_corr_) was calculated from the averaged rotational diffusion constant. A more detailed description of the spectra simulation is provided in Appendix A.

## 3. Results

### 3.1. Turbidimetry

**Figure 1:**
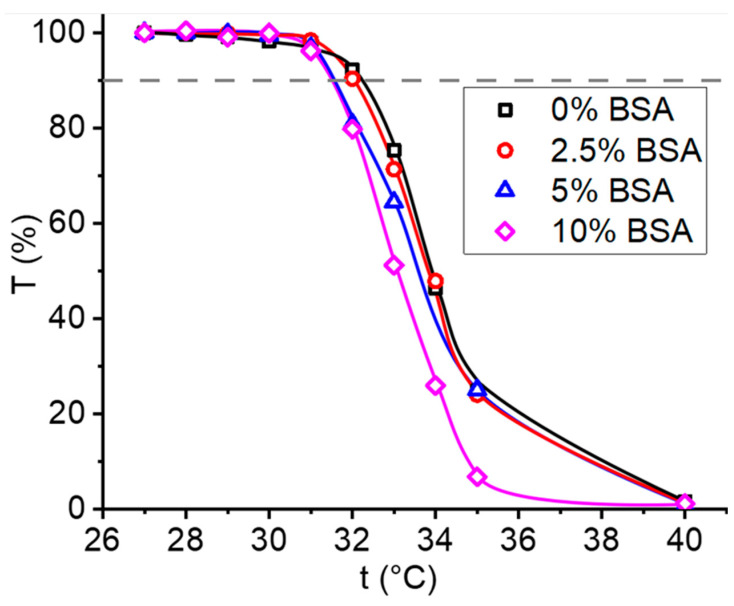
Turbidimetry curves for 1 wt% PNIPAM solutions with different BSA contents: 0 wt% BSA (black squares), 2.5 wt% BSA (red circles), 5 wt% BSA (blue triangles), and 10 wt% BSA (magenta rhombi). The dashed line represents 90% transmittance, where the *T*_cp_ was measured. All error bars are within the data points drawn. The lines are guides for the eye.

The cloud point of the 1 wt% PNIPAM aqueous solution determined by step-wise turbidimetry was equal to 32 °C, which is consistent with that previously obtained by DSC and by turbidimetry with linear heating [25]. To verify that the transmittance of the BSA solution did not change at the studied temperatures, the transmittance temperature dependence was also recorded for the 10 wt% BSA aqueous solution without PNIPAM. Transmittance curves for the PNIPAM solution and the 10 wt% BSA solution are given in the Supplementary Materials (Figure S2). The turbidity curves for a series of solutions containing 1 wt% PNIPAM and 0, 2.5, 5, and 10 wt% BSA are presented in Figure 1. The 2.5 wt% concentration of the BSA solution corresponds to the same value of bovine serum [33]. It can be seen that in the 10 wt% BSA/1 wt% PNIPAM solution, the *T*_CP_ was 1 °C lower than in the corresponding PNIPAM solution. Additionally, significant dependence of the transmittance on the protein concentration was observed within the range of 33–35 °C; the transmittance diminished with the increasing protein concentration. For instance, when heating to 33 °C, the transmittance dropped to 75% in the 1 wt% PNIPAM solution compared to 51% in the 1 wt% PNIPAM, 10 wt% BSA solution. Therefore, BSA facilitated an increase in the fraction of collapsed polymer chains or led to the formation of slightly soluble polymer–protein aggregates.

### 3.2. EPR Spectroscopy of SL-PNIPAM Solution

**Figure 2:**
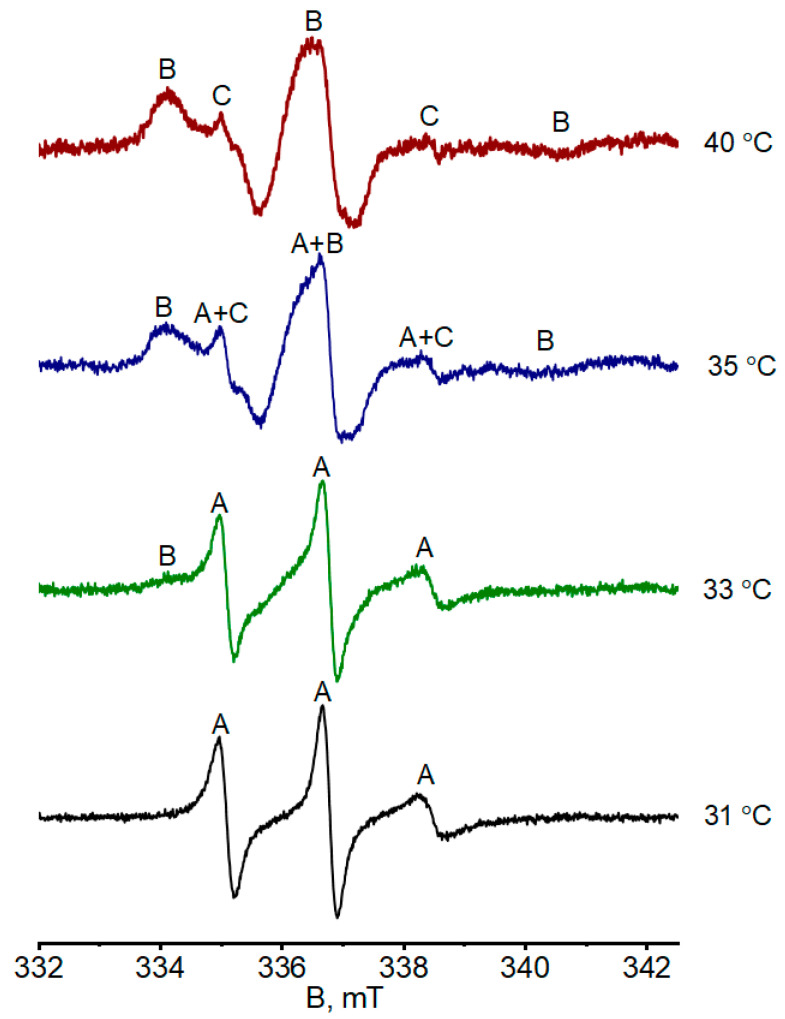
EPR spectra of 10 wt% SL-PNIPAM aqueous solution: 31 °C (black), 33 °C (green), 35 °C (blue), and 40 °C (dark red). Components of different types of paramagnetic particles are denoted in the spectra by A, B, and C letters. Type **A** particles are spin labels bound to polymer chains in the coil conformation; type **B** particles are spin labels bound to polymer chains in the globule conformation; type **C** particles are free 4-amino-TEMPO.

The EPR spectra of 10 wt% SL-PNIPAM aqueous solutions recorded at 31, 33, 35, and 40 °C are presented in Figure 2. The spectrum recorded at 31 °C (black line) is an asymmetric triplet due to the hyperfine interaction between the odd electron and the paramagnetic ^14^N nuclei with a nuclear spin I = 1. The ratio of the triplet components was 1:1.4:0.3. Such spectra are typical for slow-tumbling nitroxides groups in spi*N*-labeled polymers [27] in solutions. This signal corresponds to the label bound to freely moving polymer chains in the coil conformation, further denoted as type **A** particles. Slight heating to 32 °C led to a decrease in the amplitude of the type **A** particle spectrum and the appearance of new broad components. These components correspond to slower rotating labels bonded to collapsed polymeric chains [34], denoted as type **B** particles. Further heating to 35–40 °C resulted in the gradual full disappearance of the type **A** particle signal. The spectrum at 40 °C is close to the rigid limit spectrum [35]. The extrema separation measured as a distance between the extreme spectra components and usually denoted as 2*A*’_zz_ equaled 6.58 mT. This value approached the corresponding 2*A*_zz_ value in the spectrum of the solid SL-PNIPAM recorded at −183 °C (7.02 mT) when the motion was frozen (see Supplementary Materials, Figure S3). In the spectra recorded at 35–50 °C, low-intense sharp components came out in the spectra. They apparently correspond to a signal of the unbound free 4-amino-TEMPO molecules and are denoted as type **C** particles. Further heating to 50 °C did not cause any changes in the spectral line shape. 

**Figure 3:**
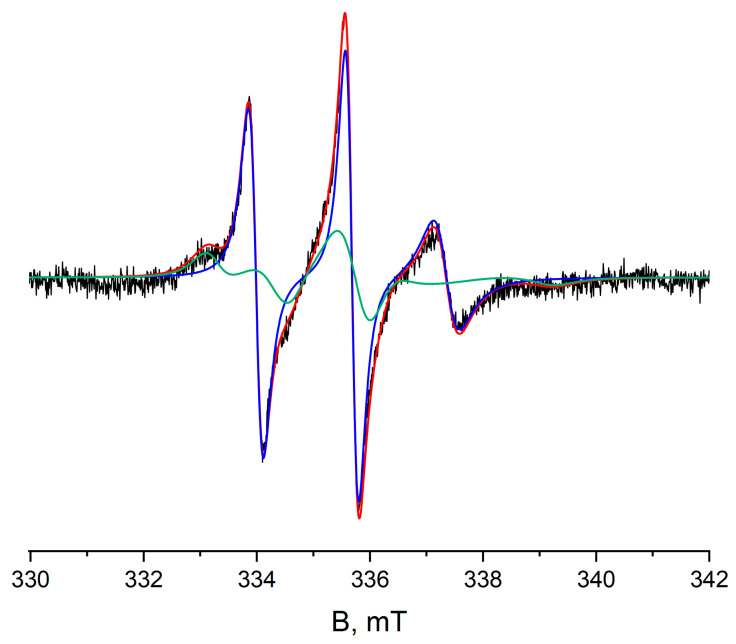
Decomposition of the spectrum of SL-PNIPAM 10 wt% aqueous solution at 33 °C. Experimental (black), simulated (red), type **A** (blue), and type **B** (green).

The spectrum at 31 °C was simulated according to the slow-motion model with the anisotropic rotation slightly hindered along the *x*-axis. The isotropic hfc constant *a*_iso_ was estimated to be 1.71 mT, and the isotropic value of the rotational correlation time *t*_iso_ was estimated to be 1.6 ns (see Supplementary Materials, Figure S4). The spectrum at 40 °C was simulated as the sum of two particles’ signals: type **B** particles in the slow-motion model with anisotropic rotation and type **C** particles in the isotropic fast-motion model. The anisotropic rotational correlation times for **B** particles were *t*_x_ = 9.2, *t*_y_ = 100.0, and *t*_z_ = 9.7 ns. The isotropic hfc constant value for **B** particles was 1.67 mT. The fraction of type **C** particles did not exceed 1%. The simulated spectrum is given in the Supplementary Materials (Figure S5). The obtained magnetic and dynamic parameters of type **A** and type **B** particles were used to fit the spectrum recorded at 33 °C as the sum of the spectra of type **A** particles and type **B** particles only by varying the particle fractions (see Figure 3). The fraction of type **B** particles at 33 °C was 53%, which means that about half of the polymer chains collapsed. The results obtained are consistent with the data of Winnik et al. [27] obtained for more diluted aqueous solutions of spi*N*-labeled PNIPAM.

### 3.3. EPR Spectra of SL-PNIPAM/BSA Solutions

**Figure 4:**
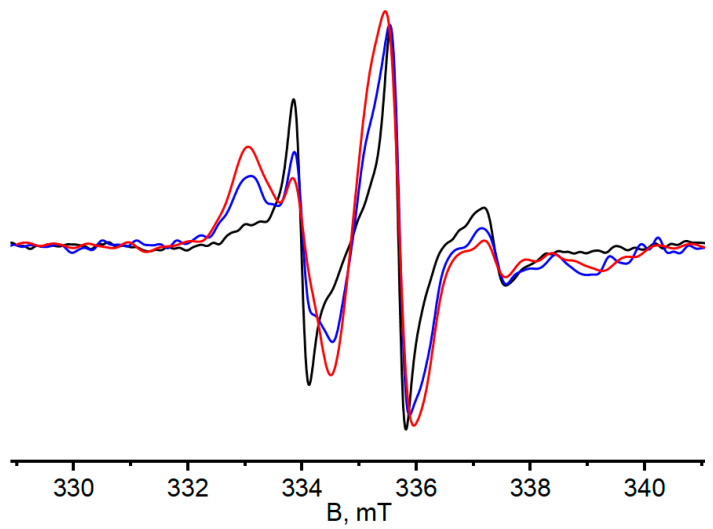
EPR spectra of SL-PNIPAM/BSA solutions at 33 °C. BSA:SL-PNIPAM = 0 (black), 1:2 (blue), 2:1 (red). Spectra were smoothed using the 5 pts FFT smoothing method for a better visual appearance. An example of the original unsmoothed spectrum is given in the Supplementary Materials (Figure S8).

The compositions of the SL-PNIPAM/BSA solutions studied by EPR spectroscopy are listed in Appendix A (Table A1). The line shapes of all these studied solutions spectra recorded below 31 °C) (Supplementary Materials, Figure S6) and above 35 °C (Supplementary Materials, Figure S7) did not depend on the BSA concentration and coincide with the spectra recorded without the protein, while the spectra in the range of 32–35 °C strongly depended on the protein concentration. A comparison of the spectra of the solutions different BSA concentrations at 33 °C with is presented in Figure 4. The amplitude of the lines corresponding to the nitroxides bound to the polymer chains in the globule conformation grows with increases in the relative concentration of BSA. All these spectra at 33 °C were simulated as the sum of the spectra of type **A** and type **B** particles with the same magnetic and dynamic parameters obtained from simulations of the SL-PNIPAM spectra in aqueous solution. The fraction of type **B** particles increased from 53% in the absence of BSA to 94% in the 10 wt% BSA solution. In other words, adding BSA to the PNIPAM solution resulted in an increased fraction of the collapsed polymer chains at 33–35 °C and may have facilitated the polymer chain collapse, while it did not affect the mobility of polymer chains below the LCST and did not change the globule structure above the LCST.

### 3.4. Spin Probe EPR

While the spin label approach allows for monitoring the mobility of polymer chains bonded to a label, the spin probe method studies the local environment and local mobility of spin probes distributed in different phases of polymer–water systems. In the case of thermoresponsive polymers solutions, the probes may be captured by collapsing globules, leading to changes in the micropolarity and microviscosity of the probe environment [25,35]. To study the effects of BSA on PNIPAM chain collapse by spin probe ESR spectroscopy, individual TEMPO/PNIPAM and TEMPO/BSA systems should be primarily investigated. Thus, two series of temperature dependencies of the TEMPO radical spectra in 1–10 wt% PNIPAM solutions and in 2.5–10 wt% BSA solutions were carried out and obtained.

PNIPAM chain collapse in 10 wt% aqueous solutions was studied by spin probe EPR spectroscopy previously [25]. It was shown that below the LCST, the spectra were triplets due to hyperfine interactions with the magnetic nuclei ^14^N, and the linewidth and amplitude ratios of the triplet components are close to the so-called fast limit [35]. In addition to the ^14^N hyperfine splittings, low-intensity satellites were observed as additional components for each of the three lines. These satellites arose from hyperfine interactions with the paramagnetic ^13^C nuclei (I = ½) in the methyl groups and carbon atoms of the piperidine ring. Above the LCST, the line shape changed dramatically due to capture of the TEMPO probe by the collapsing polymeric chains, so the observed spectra at 32–40 °C correspond to the sum of two TEMPO probe signals in different environments—in a polar aqueous solution and in low-polar globules (denoted as type **E1** probes). Particles in the polar environment that freely tumble and have magnetic (*a*_iso_ = 1.73 mT) and dynamic (*t*_corr_ = 10 ps) parameters similar to those in water are denoted as type **D** probes. 

Similar temperature changes in the spectra were observed for less concentrated solutions (1–5 wt% PNIPAM) (Figure S9, Supplementary Materials). As the concentration of the polymer decreased, the TEMPO fraction in the globules decreased, leading to a less pronounced EPR signal from these particles above the LCST. To obtain an individual EPR spectrum of the TEMPO in globules (type **E1** probes), a technique of suppressing the EPR spectrum in the presence of Cu^2+^ ions was used. This approach is based on broadening the EPR signal of radicals in solutions to alignment with the baseline due to spin–exchange interactions with copper ions [36]. According to the EPR spectra simulations, the fraction of **E1** particles dropped from 62% to 25%, while the polymer concentration fell from 10%wt to 1%wt. The simulated EPR spectrum of the TEMPO in PNIPAM globules in the presence of Cu^2+^ ions is given in the Supplementary Materials (Figure S10). The magnetic (*a*_iso_) and dynamic (*t*_corr_) parameters of the TEMPO in globules did not depend on the composition of the PNIPAM solutions (in the range of 1–10 wt%), pointing out the similar structure of PNIPAM solutions with different concentrations both below and above the LCST. Thus, the globules formed in the solutions with different PNIPAM contents exhibited similar micropolarity and microviscosity, and the polymer chain entanglement in concentrated solutions did not lead to the formation of a new types of inhomogeneities.

**Figure 5:**
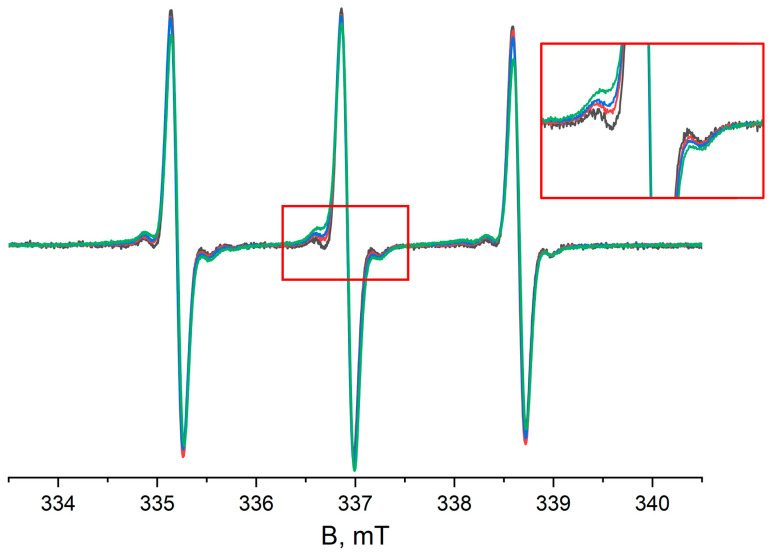
The EPR spectra of TEMPO probe in solutions with different BSA concentrations in comparison with the spectrum of TEMPO in the 10 wt% PNIPAM solution, t = 25 °C; 10 wt% PNIPAM (black), 2.5 wt% BSA (red), 5 wt% BSA (blue), and 10 wt% BSA (green). The red rectangle represents the central part of the spectra for better visual appearance. The spectrum of TEMPO in the PNIPAM solution was taken from [25].

**Figure 6:**
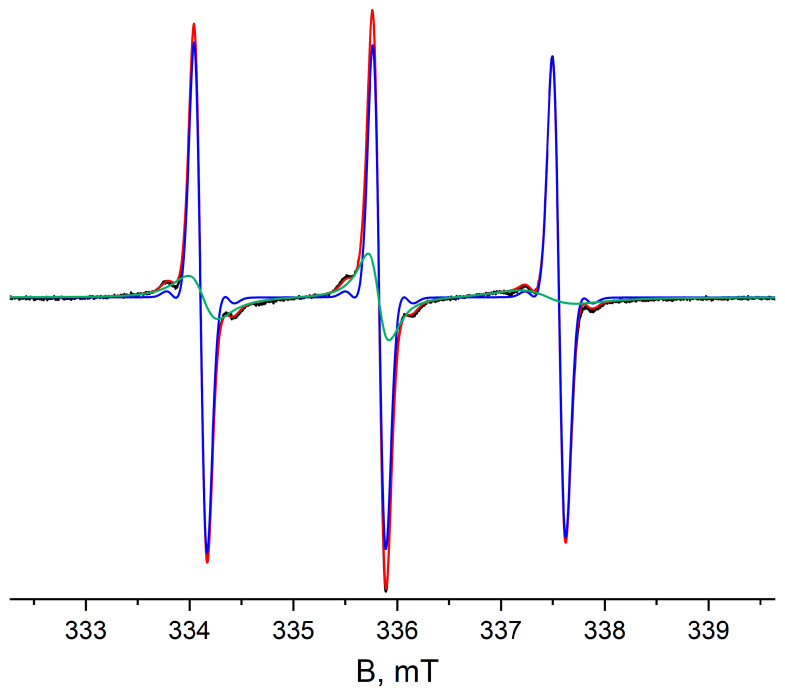
Decomposition of EPR spectrum of TEMPO in 10 wt% BSA solution, t = 25 °C. Experiment (black), simulation (red), type **D** (blue), and type **E2** (green).

**Figure 7:**
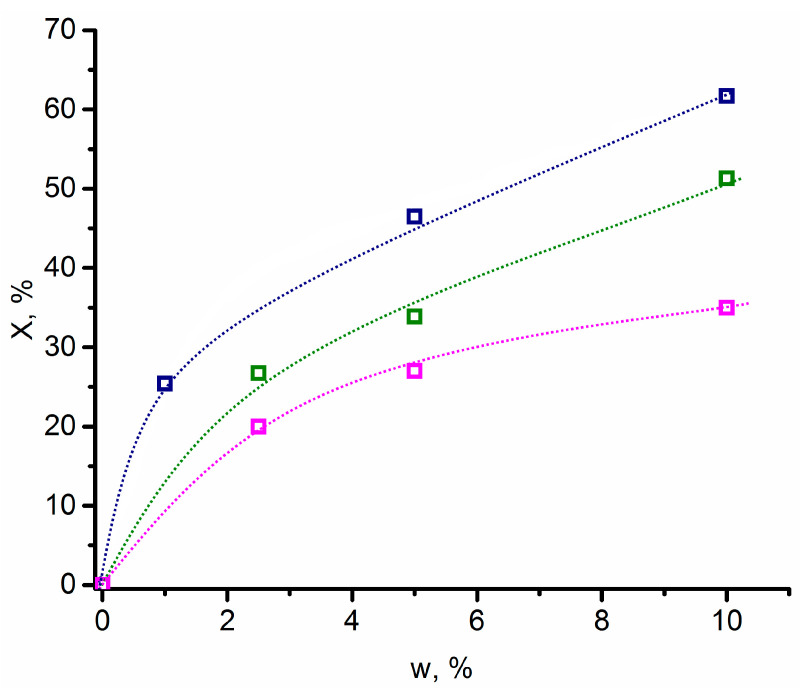
Fractions (X) of type **E1** and **E2** particles versus BSA and polymer concentration. PNIPAM solution at 40 °C (navy squares), BSA solution at 40 °C (green squares), and BSA solution at 25 °C (magenta squares). The data for the E1 particles in the 10 wt% solutions were taken from [25]. The lines are guides for the eye.

**Figure 8:**
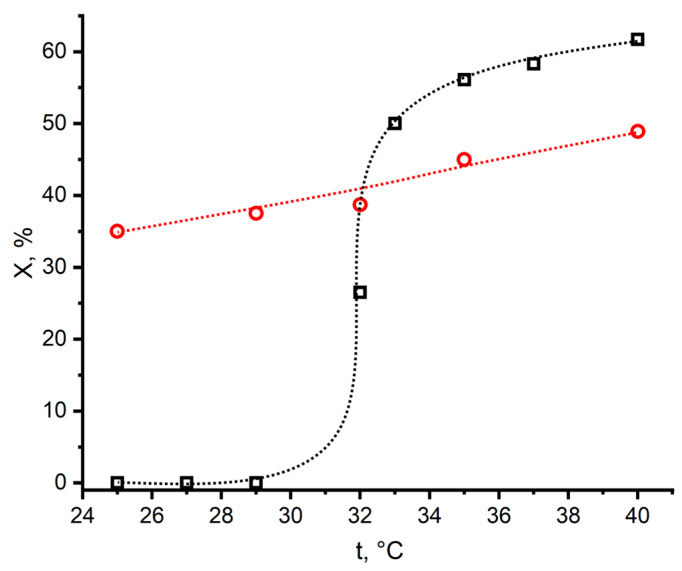
Fractions of type **E1** (black squares) and **E2** (red circles) particles in 10 wt% PNIPAM and 10 wt% BSA solutions versus temperature. The data for the E1 particles were taken from [25]. The lines are guides for the eye.

The spectra of the TEMPO in BSA solutions at room temperature differed slightly from those in the PNIPAM solutions below the LCST (see Figure 5). The spectra in the presence of BSA could not be described as an individual spectrum of one paramagnetic particle and were successfully fitted as the sum of two signals of type **D** and type **E2** particles (see Figure 6). Type **E2** corresponds to slower tumbling nitroxides and is characterized by the parameters *a*_iso_ = 1.68 mT and *t*_corr_ = 2.5 ns. The fraction of **E2** particles increased with an increasing BSA concentration (see Figure 7). Heating to 40 °C also led to an increase in the fraction of **E2** particles (to 50% in the case of the 10 wt% BSA solution; see Figure 8). 

Thus, in both the PNIPAM and BSA solutions, there are two types of spin probes with different mobilities and environment polarities. Type **D** particles in both systems are located in a polar environment and rotate with *t*_corr_ ~10 ps; their properties are similar to those in aqueous solutions. In the PNIPAM solutions, type **E1** particles appeared only above the LCST, and their fraction rapidly increased with heating. In the BSA solutions, type **E2** particles existed already at 25 °C, and their fraction linearly enhanced with heating up to 40 °C.

**Figure 9:**
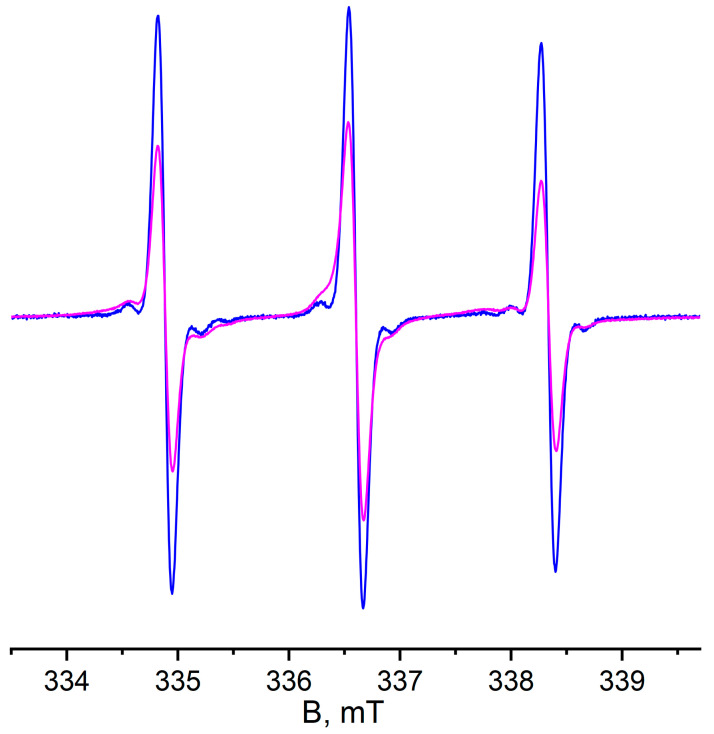
The EPR spectrum of TEMPO in the 10% PNIPAM/10% BSA solution (magenta) compared with the spectrum obtained by the summation of the individual spectra in the 10% BSA and 10% PNIPAM solutions (blue), t = 25 °C. The spectra were normalized to integral intensity.

Figure 9 demonstrates the TEMPO EPR spectrum in the 10 wt% PNIPAM/10 wt% BSA mixed solution at 25 °C. This spectrum also represents a composition of the probe spectra in different environments. Some of the probes are located in a media similar to that in the low-polar inhomogeneities of the PNIPAM solutions above the LCST, but the spectrum significantly differes from the sum of the TEMPO spectra in the PNIPAM and BSA solutions registered separately. The spectra can be simulated as the sum of the spectra of type **D** particles (in aqueous solution) and type **E3** particles (in viscous and low-polar media) with effective values of *a*_iso_ and *t*_iso_ equal to 1.60 mT and 3.8 ns, respectively (see Table 1). The fraction of slowly rotating **E3** particles was 66%. At this temperature, the PNIPAM solutions did not contain inhomogeneities, and in the BSA solutions, the fraction of the slow-mobile particles did not exceed 50% (Table 1). 

## 4. Discussion

According to the spin label data, the collapse of the PNIPAM chains continued above the LCST up to 40 °C. It appeared as an increase in the fraction of the low-mobile spin labels, referred as to **B** particles, from 52% at 33 °C to 99% at 40 °C. Similar trends were observed with IR spectroscopy by the estimation of the intermolecular H-bond fraction, which also increased up to 40 °C [37]. This indicates that the globules continued to undergo structural changes as the temperature increased above the LCST.

In the BSA solutions of TEMPO at 25–40 °C, there were two types of probes with different mobility and polarity. The first of them had an environment similar to aqueous solutions. The environment polarity of the second type of probes (**E2**) was lower than that in water, as the corresponding *a*_iso_ value (1.68 mT) was lower than the value in water (1.73 mT). The fraction of **E2** probes slightly enhanced with heating to 40 °C and increased with the protein concentration. BSA is known to form strong complexes (K = 10^4^–10^6^ M^−1^) with biologically active molecules and their derivatives, including spi*N*-labeled (SL) molecules [38]. The main role of the complex formation is hydrophobic interactions. The SL compounds in these complexes have an average rotational correlation time *t*_corr_ of about 10 ns, which also confirms the formation of strong associates. On the contrary, BSA has no effect on the EPR spectra of small, low-polar spin probes, like 4-amino-TEMPO, under these conditions, which indicates the absence of strong interactions. As obtained in the present study, changes in the TEMPO spectra in the presence of BSA with different concentrations (including physiological ones) (Figure 5) verify the formation of associates between these molecules, earlier denoted as type **E2** particles. The isotropic rotational correlation time *t*_corr_ for **E2** particles (2.5 ns) is much shorter than that for the strong complexes described before, which have an average *t*_corr_ of 10 ns. Possibly, TEMPO does not form any complexes with BSA, but it may be located in some sites or cavities with lower polarity compared to water. Such cavities are possibly formed by albumin dimers. The dimerization of BSA has numerously been confirmed by various methods, including the fluorescence resonance energy transfer method [39], small-angle X-ray scattering [40], and pulse dipolar EPR spectroscopy [41]. According to X-ray scattering data, the BSA dimer fraction reaches 15–20% in 10–40 mg/mL albumin solutions at 25 °C [40]. The approximate size of the internal cavity in the BSA dimer has been estimated to be 30 nm from the crystal structure (PDB ID: 4F5S) [42,43], and amphiphilic TEMPO particles with a ~0.7 nm diameter [44,45] may be partially located inside the protein dimer cavities due to their lower polarity. On the contrary, more polar 4-amino-TEMPO and TEMPOL molecules are preferably distributed in aqueous solutions [38]. The hypothesis was confirmed by the similarity between the temperature dependencies of low-mobile TEMPO probe fractions in the BSA solutions (Figure 8) and the BSA dimer fraction obtained by small-angle X-ray spectroscopy [40]. 

According to the turbidimetry and spin label EPR results, the presence of BSA in the PNIPAM aqueous solutions led to a decrease in the LCST and enhanced the fraction of collapsed polymer chains near the LCST. Alongside this, the presence of albumin at 2.5–10 wt% concentrations did not affect the mobility of the polymer chains at 25–40 °C, but it did influence the fraction of collapsed chains at 33–35 °C during the collapse. This effect may have been due to the diminishing of the dehydration barrier for the PNIPAM chains of adjacent water molecules by their coordination with proteins. In the PNIPAM/BSA aqueous solutions, the average environment of the TEMPO probe under ambient conditions differed from the corresponding binary PNIPAM and BSA solutions. Even at 25 °C, 66% of the TEMPO probes in these solutions, instead of 50% in the BSA solutions, rotated slower and had a more hydrophobic environment than in water, wherein the system remained transparent according to turbidimetry. Nevertheless, it could not be explained by only the increase in the BSA dimer fraction, because the effective parameters *a*_iso_ and *t*_corr,_ obtained from the modeling of the corresponding EPR spectra, were noticeably different (see Table 1). This suggests that there may be other factors at play, such as the formation of weak polymer–protein aggregates. These aggregates could form a fluctuating nanoscale network with more hydrophobic and more viscous cavities. These aggregates may be formed via H-bonds with donor atoms from both PNIPAM links and proteins.

## 5. Conclusions

In summarizing the spin label and spin probe EPR data accompanied by turbidimetry measurements on the structural features of the PNIPAM, BSA, and PNIPAM-BSA aqueous solutions at physiological temperatures, the following conclusions can be made:

- The presence of BSA in the PNIPAM aqueous solutions caused a slight decrease in the LCST and promoted polymer chain collapse in the narrow temperature region near the LCST.
- Small hydrophobic or amphiphilic molecules, e.g., drugs, may be captured by the inner cavities of BSA dimers. So, drug transport can occur not only via strong binding to proteins, but also via capturing by protein dimers.
- To predict the drug release from a PNIPAM-based drug delivery system under in vivo conditions, an estimation of the effect of BSA on drug release is necessary.

These considerations should be taken into account when studying PNIPAM-based materials for biomedical and tissue engineering purposes and when predicting drugs transport in vivo.

## Figures and Tables

**Table 1:** Several magnetic and dynamic parameters of different particles from EPR spectra simulations.

Type	t, °C	*a*_iso_, mT	*t*_x_, ns	*t*_y_, ns	*t*_z_, ns	*t*_iso_, ns	x, %
10 wt% SL-PNIPAM							
**A**	25	1.71	8.8	0.7	0.7	1.6	
**B**	40	1.67	9.2	100.0	9.7	20.7	
**C**	40	1.72	0.015	0.015	0.015	0.015	
TEMPO/1 wt% PNIPAM							
**D**	40	1.73	0.01	0.01	0.01	0.01	
**E1**	40	1.60	17.8	0.3	1.8	2.1	25%
TEMPO/10 wt% BSA							
**D**	25	1.73	0.01	0.01	0.01	0.01	
**E2**	25	1.68	20.0	0.3	2.5	2.5	50%
TEMPO/10 wt% PNIPAM + 10 wt% BSA							
**D**	25	1.73	0.01	0.01	0.01	0.01	
E3 *	25	1.60	50	0.4	2.7	3.8	66%

* Effective magnetic and dynamic parameters.

## Data Availability

The original contributions presented in the study are included in the article/Supplementary Materials, further inquiries can be directed to the corresponding author/s.

## Supplementary Materials

The following supporting information can be downloaded at: https://www.mdpi.com/article/10.3390/polym16101335/s1, Figure S1: SEC data for SL-PNIPAM, Figure S2: Turbidimetric data for 1 wt% PNIPAM solution (red circles) and 10 wt% BSA solution (black squares). The dash line is 90% transmittance, where Tcp was measured. All error bars are within the data points drawn. The lines are guides for the eye, Figure S3: EPR spectrum of SL-PNIPAM powder, t = −183 °C. Azz = 3.51 mT, rmsd = 0.0090. Experimental (black), simulation (red), Figure S4: Simulation of EPR spectrum of 10 wt% SL-PNIPAM aqueous solution, t = 31 °C. aiso = 1.71 mT, tiso = 1.6 ns, rmsd = 0.0053. Experimental spectrum (black), simulation (red); Figure S5: Simulation of EPR spectrum of 10 wt% SL-PNIPAM aqueous solution, t = 40 °C. aiso = 1.67 mT, tx = 9.2, ty = 100.0, tz = 9.7 ns, rmsd = 0.0051. Experimental (black), simulation (red), Figure S6: EPR spectra of SL-PNIPAM solutions, t = 31 °C. 0 wt% BSA (black), 10 wt% BSA (magenta), Figure S7: EPR spectra of SL-PNIPAM solutions, t = 40 °C. 0 wt% BSA (black), 10 wt% BSA (magenta), Figure S8: Smoothed EPR spectrum of 5 wt% SL-PNIPAM solution in presence of 10 wt% BSA, t = 33 °C. Experimental (black), smoothed (red), Figure S9: EPR spectra of TEMPO probe in solutions with different PNIPAM concentrations, t = 40 °C. 1 wt% PNIPAM (wine), 5 wt% PNIPAM (green), 10 wt% PNIPAM (navy), Figure S10: Simulation of EPR spectrum of TEMPO in 1 wt% PNIPAM solution in the presence of Cu^2+^ ions, t = 60 °C. aiso = 1.60 mT, tx = 17.8, ty = 0.3, tz = 1.8 ns. Experimental (black), simulation (red).

## Appendix A

### Appendix A.1. EPR Spectra Simulation Details

All CW EPR spectra simulations were performed with the MATLAB (v. R2023a) program package employing the EasySpin (v. 5.2.35) toolkit [30]. Only original unsmoothed spectra were used in the simulations. The quality of fitting was controlled by the calculation of the root-mea*N*-square deviation for the different spectra. The root-mea*N*-square deviation obtained from the simulations of all fitted spectra was less than 1%. The spectral and dynamic parameters were obtained by fitting the simulated EPR spectra to the experimental data using least-squares fitting algorithms [46]. Line broadening was defined in relation to the isotropic rotational correlation time, as well as via additional convolution of Gaussian and Lorentz line broadening. In the simulations, only the *g*_xx_ and *A*_zz_ components were varied as magnetic parameters, as they are more sensitive to the environment. The isotropic *g*_iso_ and *a*_iso_ values were calculated as averages of the diagonal elements:

(A1) $$g_{iso}=\frac{1}{3}\left(g_{xx}+g_{yy}+g_{zz}\right)$$

(A2) $$a_{iso}=\frac{1}{3}\left(A_{xx}+A_{yy}+A_{zz}\right)$$

The rotational correlation times along the different axes and the line widths could only be varied independently due to their simultaneous influence on the line shape. The averaged (isotropic) correlation time *t*_iso_ was calculated from the isotropic rotational diffusion constant using the following equation, as recommended in [32]:

(A3) $$t_{iso}=\frac{1}{6\sqrt[{3}]{D_{xx}D_{yy}D_{zz}}}$$

The spectrum of the SL-PNIPAM powder was simulated using the ‘pepper’ function in EasySpin. Magnetic parameters of 4-aminoTEMPO in PEO/PPO matrix [47] were taken as an initial guess. The spectra of the SL-PNIPAM solutions were simulated using the ‘chili’ function in EasySpin. Particles of three types were used in the simulations. Type **A** particles correspond to labels covalently bound to polymer chains in the coil conformation. Type **B** particles correspond to labels covalently bound to polymer chains in the globule conformation. Type **C** particles correspond to unbound free label molecules. Spectra below the LCST were simulated as single type **A** particles. Spectra recorded at 33–35 °C were simulated as the sum of type **A** and type **B** particles. Spectra recorded at 40 °C were simulated as the sum of type **B** and type **C** particles. Even though type **C** particles always exist in solutions, they were not clearly observed in the spectra up to 40 °C due to the closeness of their magnetic parameters to type **A** labels and the overlap with their signal. The initial values for the magnetic parameters for all particle types were taken from the spectrum of 4-amino-TEMPO in aqueous media [38]. The molar fraction of the particles of each type (χ) was calculated from the weight values of these particles’ spectrum in the full EPR spectrum, using the following equation:

(A4) $$\chi_{i}=\frac{w\left(i\right)}{\sum_{k}w\left(k\right)}$$

The spectra of the TEMPO radicals in solutions (type **D** + type **E1**, **E2**, **E3**) were simulated using the ‘chili’ function in EasySpin. In addition to the splittings on the ^14^N nuclei for the type **D** probes, isotropic hyperfine splittings on the ^13^C isotopes in six CH_3_-groups with *a*_iso_ = 0.54 mT were accounted for. To simplify the modeling procedure, we changed the abundances for carbon isotopes instead of adding six paramagnetic nuclei. The initial parameters for the type **D** probes were taken from the spectra simulations of the TEMPO radical in water at different temperatures [25]. The initial parameters for the type **E1**, **E1**, **E3** probes were taken from the spectra simulations of the TEMPO radical in the polymer solutions recorded in the presence of Cu^2+^ ions at 50 °C [25].

The uncertainties for the simulated parameters were calculated as recommended in [48], and they were estimated as follows:

*a*_iso_ ± 0.01 mT (A5)

*g*_iso_ ± 0.00003 (A6)

χ ± 1% (A7)

Uncertainties in the rotational correlation time depend on its absolute value, so, for times in the range of 10–100 ns, the uncertainty was about 1 ns; for times in the order of nanoseconds, the uncertainty was about 0.1 ns.

### Appendix A.2. SL-PNIPAM/BSA Solutions EPR Spectra

**Table A1:** Concentrations of studied SL-PNIPAM/BSA solutions.

N	w(SL-PNIPAM), wt%	w(BSA), wt%	w(PNIPAM)/w(BSA)
1	10	0	N/A
2	10	2.5	4:1
3	5	2.5	2:1
4	5	10	1:2
