# Supplementary material for: Bovine Serum Albumin Effect on Collapsing PNIPAM Chains in Aqueous Solutions: Spin Label and Spin Probe Study
Source: Polymers (Basel). 2024 May 9;16(10):1335. doi: 10.3390/polym16101335 (PMC11124808; doi:10.3390/polym16101335)
Supplement: Supplementary file 1 [file polymers-16-01335-s001.zip › polymers-2964620-supplementary.pdf]

Supplementary Materials

Bovine serum albumin effect on collapsing PNIPAM chains in aqueous solutions: spin label and spin probe study

Georgii A. Simenido, Ekaterina M. Zubanova, Evgenii A. Ksendzov, Sergei V. Kostjuk, Peter S. Timashev and Elena N. Golubeva

Size Exclusion Chromatography data for SL-PNIPAM

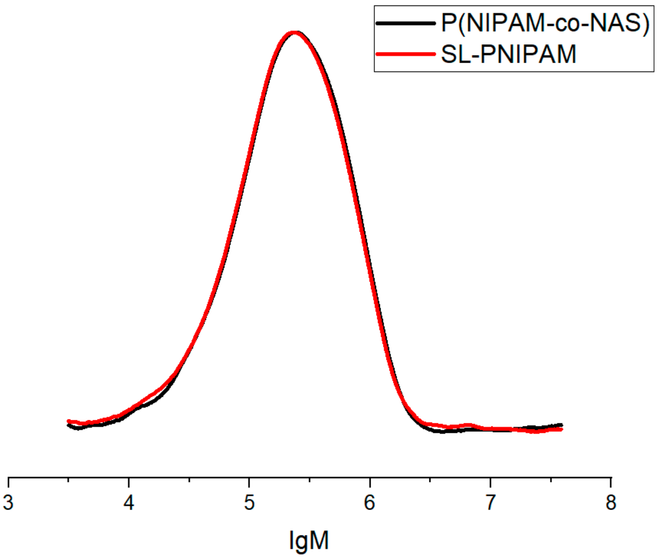

Figure S1. SEC curves for P(NIPAM-co-NAS) (black) and SL-PNIPAM (red).

Turbidimetry

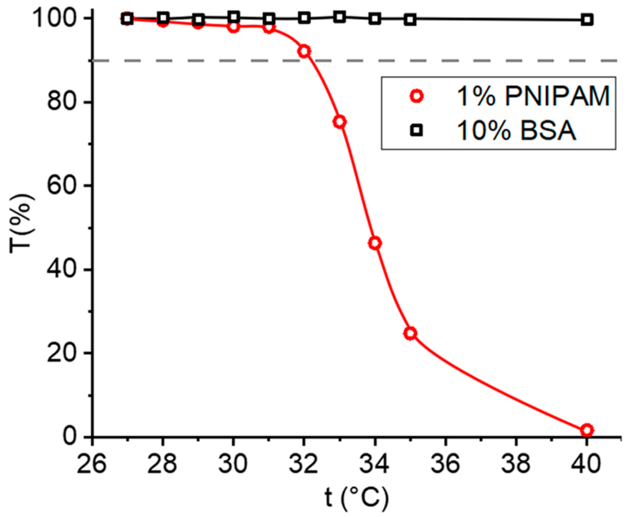

Figure S2. Turbidimetric data for 1 wt% PNIPAM solution (red circles) and 10 wt% BSA solution (black squares). The dash line is 90% transmittance, where T_{cp} was measured. All error bars are within the data points drawn. The lines are guides for the eye.

SL-PNIPAM EPR spectra

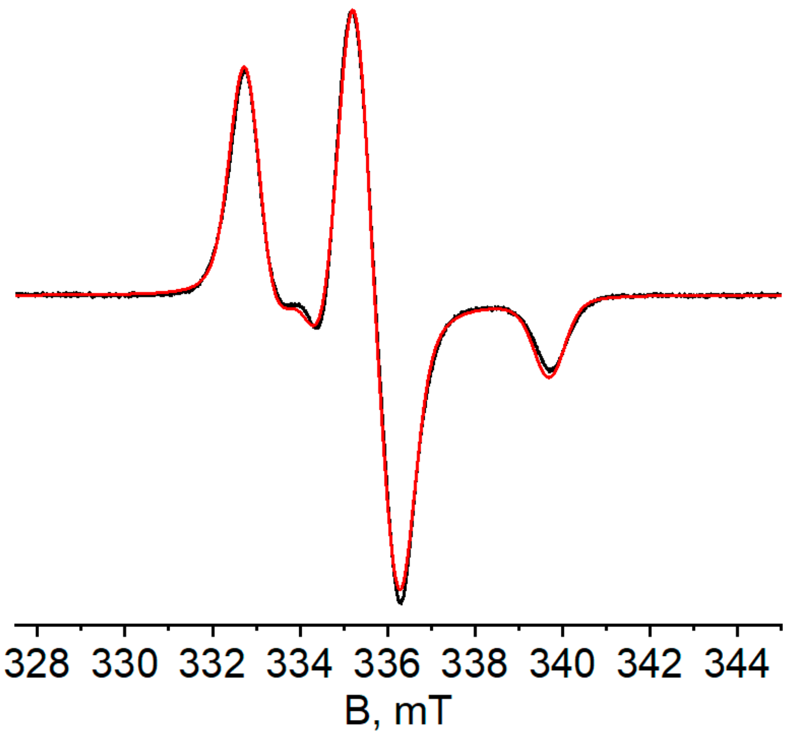

Figure S3. EPR spectrum of SL-PNIPAM powder, t = -183 °C. A_{zz} = 3.51 mT, rmsd = 0.0090. Experimental (black), simulation (red).

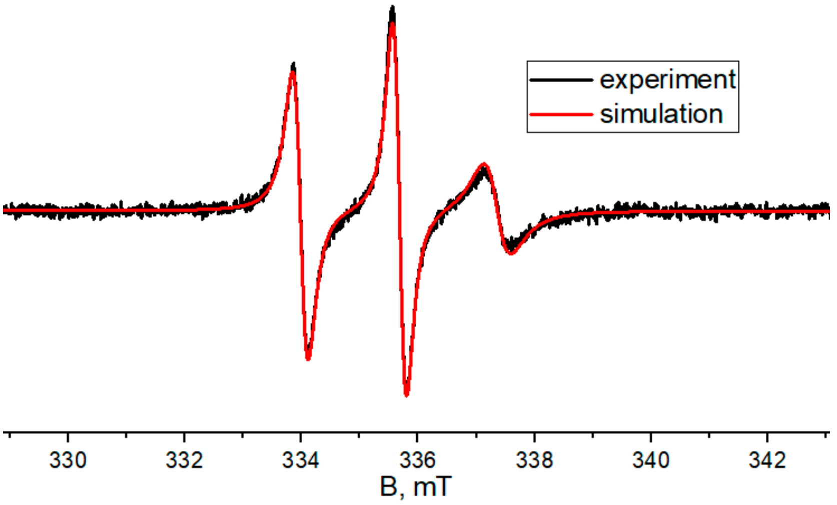

Figure S4. Simulation of EPR spectrum of 10 wt% SL-PNIPAM aqueous solution, t = 31 °C. a_{iso} = 1.71 mT, t_{iso} = 1.6 ns, rmsd = 0.0053. Experimental spectrum (black), simulation (red).

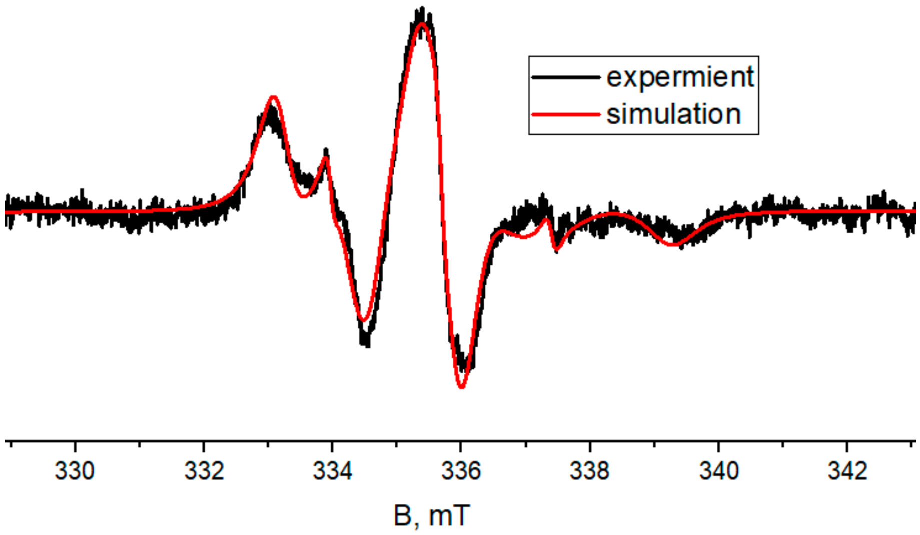

Figure S5. Simulation of EPR spectrum of 10 wt% SL-PNIPAM aqueous solution, t = 40 °C. a_{iso} = 1.67 mT, t_x = 9.2, t_y = 100.0, t_z = 9.7 ns, rmsd = 0.0051. Experimental (black), simulation (red).

SL-PNIPAM/BSA Solutions EPR Spectra

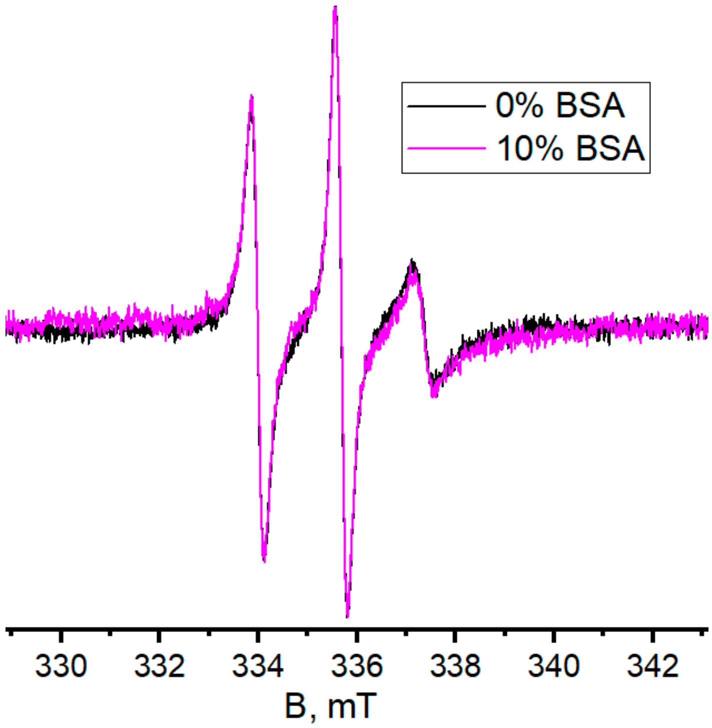

Figure S6. EPR spectra of SL-PNIPAM solutions, t = 31 °C. 0 wt% BSA (black), 10 wt% BSA (magenta).

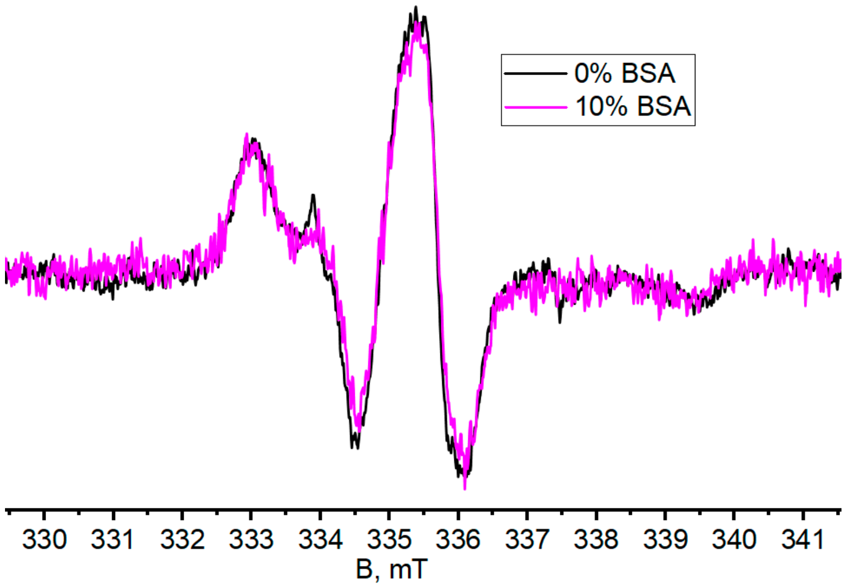

Figure S7. EPR spectra of SL-PNIPAM solutions, t = 40 °C. 0 wt% BSA (black), 10 wt% BSA (magenta).

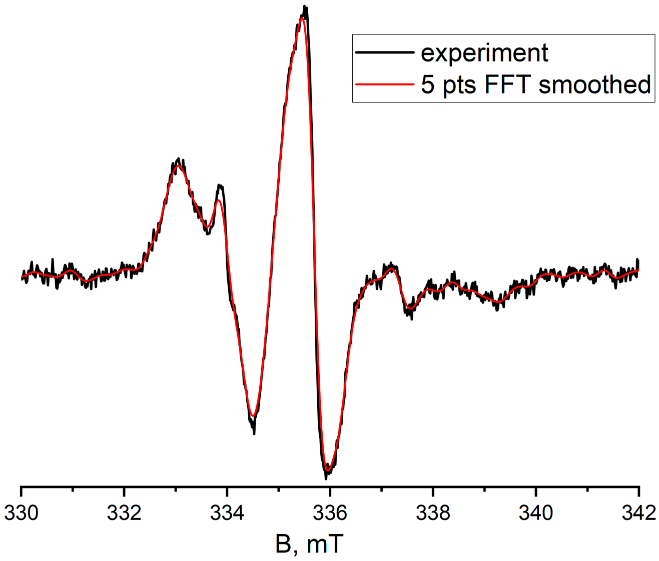

Figure S8. Smoothed EPR spectrum of 5 wt% SL-PNIPAM solution in presence of 10 wt% BSA, t = 33 °C. Experimental (black), smoothed (red).

TEMPO / PNIPAM EPR spectra

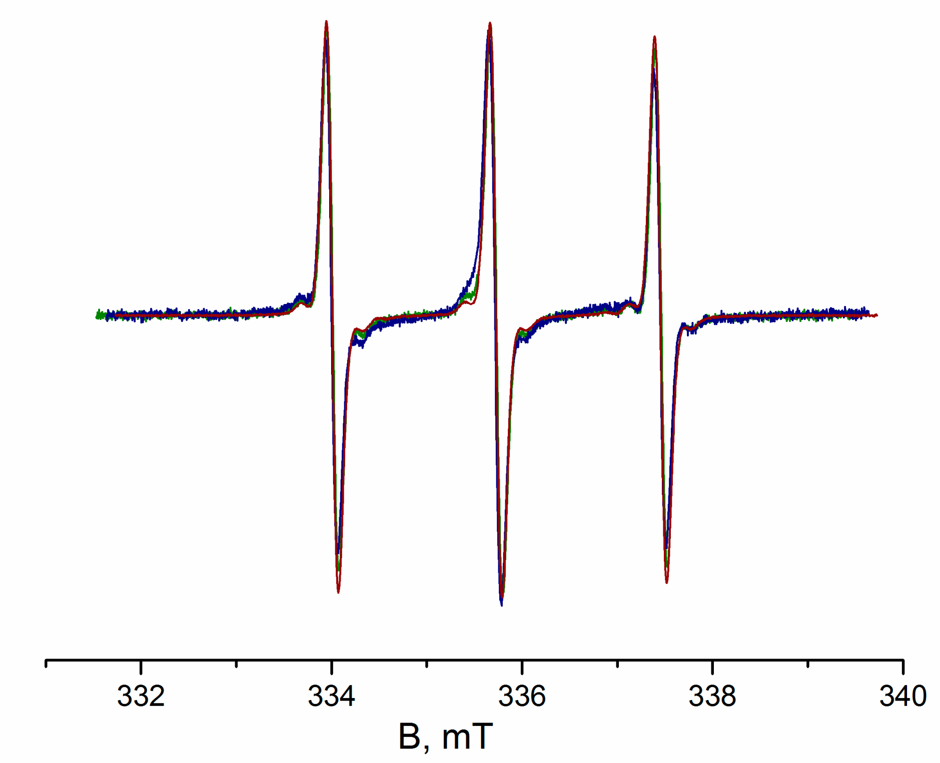

Figure S9. EPR spectra of TEMPO probe in solutions with different PNIPAM concentrations, t = 40 °C. 1 wt% PNIPAM (wine), 5 wt% PNIPAM (green), 10 wt% PNIPAM (navy).

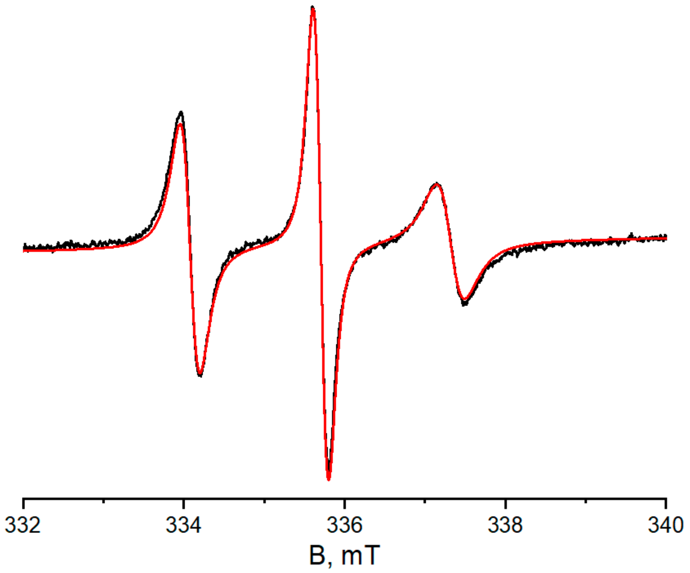

Figure S10. Simulation of EPR spectrum of TEMPO in 1 wt% PNIPAM solution in the presence of Cu^{2+} ions, t = 60 °C. a_{iso} = 1.60 mT, t_x = 17.8, t_y = 0.3, t_z = 1.8 ns. Experimental (black), simulation (red).
